# Measuring the disease burden of seasonal influenza in Germany 2015 - 2020 using the incidence-based disability-adjusted life years (DALYs)

**DOI:** 10.1186/s12879-025-10613-2

**Published:** 2025-03-26

**Authors:** Marin Stapic, Ricarda Sophia Schulz, Elena Tamayo-Cuartero, Tobias Kurth, Ralph Brinks

**Affiliations:** 1 Institute of Public Health, Charité - Universitätsmedizin Berlin, Berlin, Germany; 2 Chair of Medical Biometry and Epidemiology, Faculty of Health/School of Medicine, Witten, Germany

**Keywords:** Influenza, Disability-adjusted life years, Infectious disease surveillance, Communicable diseases, Burden of disease, Population health

## Abstract

**Background:**

Seasonal influenza can lead to severe complications and death, resulting in high disease burden each year. The European Centre for Disease Prevention and Control introduced the Burden of Communicable diseases in Europe (BCoDE) project, quantifying the disease burden of infectious diseases in disability-adjusted life years (DALY). DALYs for influenza exceed those of Tuberculosis, HIV, and Invasive pneumococcal disease. As data on disease burden are limited, this study aims to calculate the seasonal influenza burden for Germany between 2015 and 2020.

**Methods:**

The BCoDE-toolkit developed by the European Centre for Disease Prevention and Control was used, calculating country-specific DALYs. Information on incidence, population data, and underestimation were taken from the Robert Koch-Institute and the Federal Statistical Office of Germany. Outcome trees were created based on information from a rapid review and previous publications. Baseline, lower-bound and upper-bound scenarios were developed to assess the disease burden under varying conditions.

**Results:**

Estimates range from 127,100 DALYs (153 DALYs per 100,000 population) and 1,171,115 DALYs (1,414 DALYs per 100,000 population) depending on the scenario and year examined. The main contributors to the disease burden are sequelae, primarily pneumonia, encephalitis, and myocarditis. The highest burden estimates are observable for infants, children under the age of five and the elderly.

**Conclusions:**

Using a composite health measure like DALY can offer valuable insight into a disease’s impact on population health. Our results indicate a high disease burden due to seasonal influenza in Germany, indicating further research into complication rates, underestimation, and intervention programs for vulnerable populations, e.g., vaccination in infants, children under age of five and elderly population.

**Supplementary Information:**

The online version contains supplementary material available at 10.1186/s12879-025-10613-2.

## Background

The European Centre for Disease Prevention and Control (ECDC) launched the Burden of Communicable Disease in Europe (BCoDE) project intending to develop and use a methodology framework to measure the disease burden of communicable diseases in the European Union (EU) and European Economic Area (EEA). Using the incidence-based DALY approach to estimate the burden of 31 communicable diseases between 2009 and 2013, the authors of the BCoDE project report a total burden of 275 DALYs per 100,000 population, of which seasonal influenza accounted for 30%, corresponding to 81.8 DALYs per 100,000 population [1]. Other infectious diseases such as Tuberculosis and HIV/AIDS can cause between 8 DALYs per 100,000 population and 787 DALYs per 100,000 population, depending on the region and country (Table 1).

**Table 1:** Burden of disease of various infectious diseases - Total DALYs (and DALYs per 100,000 population)

Region/Country	Tuberculosis	HIV/AIDS	Acute Hep. B	Measles	LRI	URI
Western Europe	67,266(15)	185,940(43)	5,573(1.28)	180(0.04)	1,802,174(413)	363,202(83)
France	14,932(23)	26,242(40)	928(1.40)	20(0.03)	234,856(355)	55,215(83)
Sweden	1,237(12)	1,273(12)	85(0.83)	0.8(0.01)	30,928(303)	9,033(88)
United Kingdom	9,000(13)	20,297(30)	662(0.98)	67(0.1)	474,724(706)	59,146(88)
Germany	10,045(12)	23,320(27)	708(0.83)	49(0.06)	317,689(374)	67,879(80)
Central Europe	69,881(61)	24,404(21)	1,882(1.65)	625(0.55)	599,840(525)	73,395(64)
Albania	317(12)	83(3)	32(1.18)	121(4.44)	12,404(456)	2,134(78)
Hungary	1,585(16)	1,620(17)	149(1.54)	1(0.01)	22,091(228)	6130(63)
Poland	17,011(44)	7,631(20)	416(1.08)	7(0.02)	206,880(538)	25,432(66)
Eastern Europe	539,277(257)	1,452,867(692)	6,682(3.18)	333(0.16)	1,423,084(678)	159,882(76)
Estonia	877(67)	2,041(156)	12(0.89)	0.2 (0.01)	4,934(376)	875(67)
Lithuania	5,489(196)	2,859(102)	32(1.15)	4(0.1)	12,351(442)	1,863(67)
Ukraine	168,093(382)	346,668(787)	2,751(6.25)	276(0.63)	298,090(677)	32,807(74)
North America	28,251(8)	433,184(119)	2,901(0.80)	69(0.02)	1,339,934(368)	379,345(104)
USA	25,031(8)	415,325(127)	2,273(0.69)	50(0.02)	1,228,694(375)	343,443(105)
Canada	3,145(9)	17,763(49)	626(1.72)	5(0.01)	110,971(304)	35,839(98)
Greenland	75(133)	88(157)	0.94(1.67)	14(24)	248(441)	58(102)

*DALYs* Disability-Adjusted Life Years, *LRI* Lower respiratory infection, *URI* Upper respiratory infection

Source: GBD 2019 [2]

Similar approaches, wherein the disease burden of seasonal and pandemic influenza was studied, report 2.6 DALYs per 100,000 population to 300 DALYs per 100,000 population [3–6]. While attempts to measure the disease burden due to seasonal influenza have been undertaken internationally by the WHO and ECDC, in Europe, efforts to estimate the national disease burden for a single country have been mainly limited to the Netherlands [4–6]. In Germany, seasonal influenza has the highest incidence rates among all infectious diseases, ranging from 115 to 330 cases per 100,000 population in recent years [7]. To date, the only national disease burden estimates for seasonal influenza in Germany, expressed in DALY, have been calculated by Plass et al., using the BCoDE methodology. In addition to death and recovery, permanent disabilities due to sepsis, acute respiratory distress syndrome (ARDS), and otitis media were included as sequelae. In their modeling approach, Plass et al. report 40.2 DALYs per 100,000 population. However, the disease burden might be underestimated, as sequelae following a symptomatic influenza infection besides death were limited to three long-term disabilities only [8]. Further sequelae that can occur after a symptomatic infection, are pneumonia, bronchitis and its chronic manifestations, myocarditis, encephalitis, and sinusitis [9]. Therefore, the aim of the present study is to estimate the annual DALYs caused by seasonal influenza from 2015 to 2020 in Germany based on the incidence-based DALY approach using the BCoDE-Toolkit developed by the ECDC, taking more accurate estimates of sequelae into account.

## Methods

The methodologies used were a rapid review to identify sequelae and their transition probabilities, followed by the design of an outcome tree for three scenarios, examining disease burden calculations under varying conditions. Finally, the BCoDE-toolkit was employed to analyze disease burden for all years and scenarios.

### Rapid review

**Fig. 1:**
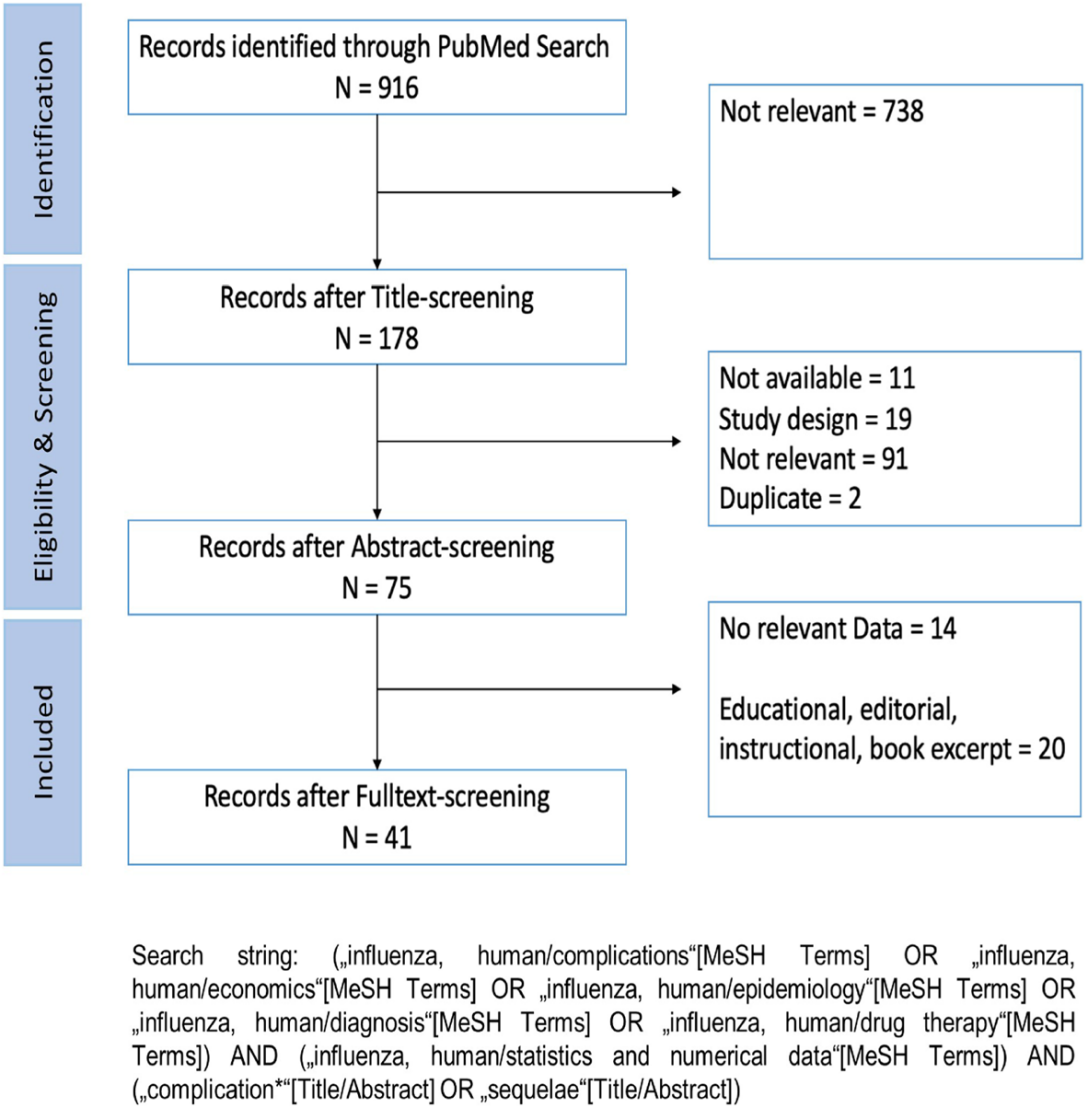
PRISMA flow diagram

Following the Cochrane Rapid Reviews Methods Group guidelines, a rapid review of influenza-related complications and transition probabilities was conducted. Screening of abstract, title and full text was conducted by three researchers (MS, RSS, ETC) independently. Studies published since 2000 were included using the PubMed database. Keywords for influenza-related complications were identified and used in combination with MeSH terms and Boolean operators (Fig. 1). All searches were conducted in October 2021.

Included studies were in German or English and examined complications following a symptomatic influenza infection, providing estimates on complication rates or probabilities. Eligible study designs were cross-sectional, case-control, cohort studies, and systematic reviews and meta-analyses. Editorials, conference abstracts, presentations, book excerpts, commentaries, case studies, case series and animal studies were excluded. Studies not published in peer-reviewed journals or lacking full text were also excluded. Complication probabilities were synthesized using population size weighting, commonly used in meta-analysis, wherein a summary estimate is calculated to address sample size differences [10] (Supplementary Table 1, Additional file 1).

### Incidence-based DALY approach and BCoDE-Toolkit

**Fig. 2:**
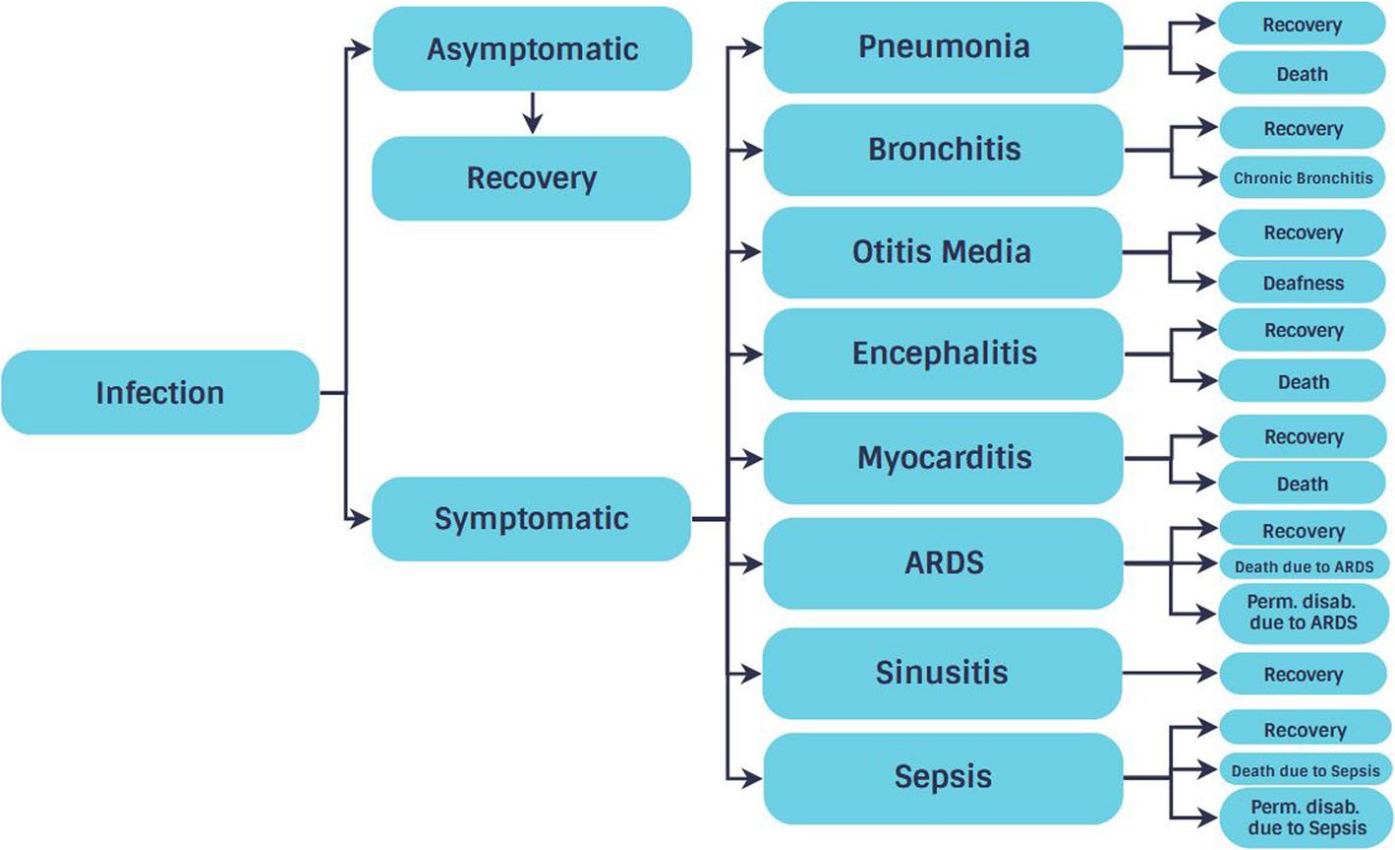
Outcome tree

The methodology for calculating disease-specific DALYs was introduced by Murray et al. in the GBD project. One DALY represents one year of healthy life lost, and is the sum of the years lost due to premature death (YLL) and the years lived with disability (YLD) [11]. Therefore, the DALY is a composite health measure, considering a disease’s impact beyond a single metric as frequency or mortality. The YLL is determined by multiplying fatal cases by the remaining life expectancy at the age of death. YLD is calculated by multiplying the duration of illness by a severity weight that represents the disability’s severity, aggregated across all cases and health outcomes [4]. In addition to death and recovery, sequelae like pneumonia, bronchitis, otitis media, encephalitis, myocarditis, ARDS, sepsis, sinusitis, and their complications, including long-term disability and death, are considered [9, 12] (Fig. 2).

The BCoDE-Toolkit, developed by the ECDC, is a publicly available software tool for calculating disease burden. Input data includes incidence, population, underestimation, life expectancy by age and sex, and parameters such as probability of health outcomes, illness duration, disability weight, case coverage, and age distribution. Output data includes total and sequelae-specific YLLs, YLDs, and DALYs, both in absolute numbers and per 100,000 population [13, 14]. Results of previous efforts that investigated the disease burden of seasonal or pandemic influenza were compared to the present modeling approach (Table 2).

**Table 2:** Influenza burden - Total DALYs, YLL, YLD, DALYs per 100,000, and DALYs per case in previous modeling approaches

Author	Region/Country	Total DALY	Total YLL	Total YLD	DALYs per 100,000	DALYs per Case
Van Lier et al. (2007) [3] Seasonal Infl.	EU	2,161	1,293-4,654	2,808-16,342	3.7	N/A
Wielders et al. (2012) [4] Pandemic Infl.	Netherlands	4,827-7,146	1,468-2,216	2,246-4,930	29–43	N/A
Plass et al. (2014) [8] Seasonal Infl.	Germany	33,116	17,077	16,040	40.2	0.03
Van Lier et al. (2016) [5] Seasonal Infl.	Netherlands	8,670	4,580	4,090	N/A	0.026
Cassini et al. (2018) [1] Seasonal Infl.	EU/EEA	411,334	383,999	27,334	81.8	0.01

*DALYs* Disability-Adjusted Life Years, *YLL* Years of Life Lost due to Premature Mortality, *YLD* Years lived with Disability, *EU* European Union, *EEA* European Economic Area

### Underestimation and uncertainty

Multiplication factors were applied to surveillance data reported by the Robert Koch-Institute (RKI) to correct for underestimation. Age- and sex-specific multiplication factors were derived from the seasonal influenza reports published by the “Arbeitsgemeinschaft Influenza” (AGI) for Germany [15–19]. These factors were based on a modeling approach by An der Heiden et al., calculating medically attended acute respiratory infections attributable to influenza in Germany [20] (Supplementary Table 2, Additional file 1). Uncertainty in parameter accuracy was accounted for using uniform and Program Evaluation and Review Technique (PERT) distributions. Furthermore, 95% uncertainty intervals (UIs) were calculated for all output parameters. For each model, 10,000 iterations of the Monte Carlo simulation were run. In line with the WHO methodology for global burden of disease estimates published in 2020, discounting was not considered [21].

### Scenario analysis

In addition to a baseline scenario, lower-bound and upper-bound scenarios were developed to examine the range of DALYs calculated under varying conditions. Transition probabilities, redistributions, and disability weights were assumed to remain constant from 2015 to 2020 for each scenario. Incidence data on influenza were obtained from SurvStat@RKI2.0 [7], an online query tool provided by the Robert Koch-Institute. Population size and life expectancy data were extracted from the GENESIS-ONLINE query offered by the Federal Statistical Office of Germany [22] (Supplementary Table 3, Additional file 1). Lower transition probabilities were assumed for pneumonia, otitis media, and encephalitis in the lower-bound scenario, along with lower death probabilities for myocarditis and ARDS. In the upper-bound scenario, higher transition probabilities for ARDS were derived from the AGI seasonal influenza report 2018/2019, and higher death probabilities were assumed based on the rapid review estimate [23–26] (Supplementary Table 4, Additional file 1).

## Results

### Rapid review

The rapid review conducted in the present study yielded information on further sequelae that can occur after a symptomatic infection. Sequelae that were considered for the outcome tree in addition to ARDS, sepsis and otitis media are pneumonia [9, 12, 27–59], bronchitis and its chronic manifestations [9, 27, 29, 30, 36, 37, 42, 44, 45, 48, 54, 57], myocarditis [9, 27, 33, 46, 48, 50, 58], encephalitis [9, 31, 36, 45–47, 49, 57, 60–62], and sinusitis [9, 27, 29, 32, 33, 36, 38, 45, 48, 54, 57].

### Seasonal influenza burden

The total burden attributed to seasonal influenza varies across scenarios and seasons, with a minimum of 127,100 DALYs and a maximum of 1,171,115 DALYs. Highest and lowest DALY estimates across every year and season are 696,543 DALYs and 276,282 DALYs for the baseline scenario, 319,756 DALYs and 127,100 DALYs for the lower-bound scenario, as well as 1,171,115 DALYs and 439,651 DALYs for the upper-bound scenario, respectively. Analogous to the total burden, estimates for the influenza burden per 100,000 population and DALYs per case also vary depending on season and scenario, ranging from 153 DALYs per 100,000 population (0.04 DALYs per case) to 1,414 DALYs per 100,000 population (0.13 DALYs per case). Highest and lowest burden estimates per 100,000 population are 841 DALYs per 100,000 population (0.08 DALYs per case) and 332 DALYs per 100,000 population (0.08 DALYs per case) for the baseline scenario, 386 DALYs per 100,000 population (0.04 DALYs per case) and 153 DALYs per 100,000 population (0.04 DALYs per case) for the lower-bound scenario, along with 1,414 DALYs per 100,000 population (0.13 DALYs per case) and 529 DALYs per 100,000 population (0.12 DALYs per case) for the upper-bound scenario (Table 3). Detailed results for all years and scenarios are listed in Additional file 2.

**Table 3:** Seasonal influenza burden - Total DALYs, DALYs per 100,000 population, DALYs per case and incidence 2015–2020

Year	Scenario	Total DALYs	DALYs/100,000	DALYs/Case	Incidence
2015	Lower-bound	208,802 (208,807-213,330)	257 (251–262)	0.03 (0.03-0.03)	7,524
	Baseline	455,066 (450,591-459,505)	560 (554–565)	0.07 (0.07-0.07)	
	Upper-bound	784,257 (779,798-788,687)	965 (960–971)	0.13 (0.13-0.13)	
2016	Lower-bound	185,383 (181,545-189,201)	179 (174–183)	0.04 (0.04-0.04)	4,927
	Baseline	319,314 (315,543-323,061)	388 (383–393)	0.08 (0.08-0.08)	
	Upper-bound	510,053 (506,299–513,802)	620 (616–625)	0.13 (0.13-0.13)	
2017	Lower-bound	204,056 (199,424-208,657)	247 (241–252)	0.04 (0.04-0.04)	7,143
	Baseline	444.336 (439,747-448,876)	538 (523–543)	0.08 (0.08-0.08)	
	Upper-bound	757,136 (752,598–761,629)	917 (912–922)	0.13 (0.13-0.13)	
2018	Lower-bound	319,756 (312,630-326,879)	386 (377–394)	0.04 (0.04-0.04)	11,158
	Baseline	696,543 (689,578–703,512)	841 (832–849)	0.08 (0.08-0.08)	
	Upper-bound	1,171,115 (1,164,082-1,178,036)	1,414 (1,406-1,422)	0.13 (0.13-0.13)	
2019	Lower-bound	127,100 (123,906-130,275)	153 (149–156)	0.04 (0.03–0.04)	4,304
	Baseline	276,282 (273,139–279,387)	332 (329–336)	0.08 (0.08-0.08)	
	Upper-bound	439,651 (436,515-442,762)	529 (525–533)	0.12 (0.12-0.12)	
2020	Lower-bound	136,381 (132,716-140,039)	163 (159–168)	0.04 (0.04-0.04)	4,441
	Baseline	295,678 (292,069–299,235)	355 (351–359)	0.08 (0.08-0.08)	
	Upper-bound	453,003 (449,495-456,553)	544 (540–548)	0.12 (0.12-0.12)	

*DALYs* Disability-Adjusted Life Years, *Incidence* Incidence of acute symptomatic influenza cases per 100,000 stratum specific population

### Disease burden due to YLL and YLD

For the baseline scenarios, premature mortality due to a symptomatic infection (YLL) is the main contributor to the total DALYs observed with an average of 90% for all years. The remaining 10% are caused by years lived with disability (YLD). A similar trend is observed for both lower-bound and upper-bound estimates: while the average share of YLL and YLD for all lower-bound scenarios are 79% and 21%, respectively, YLL and YLD averages for all upper-bound scenarios are 86% and 14%.

### Disease burden due to acute infection and sequelae

**Fig. 3:**
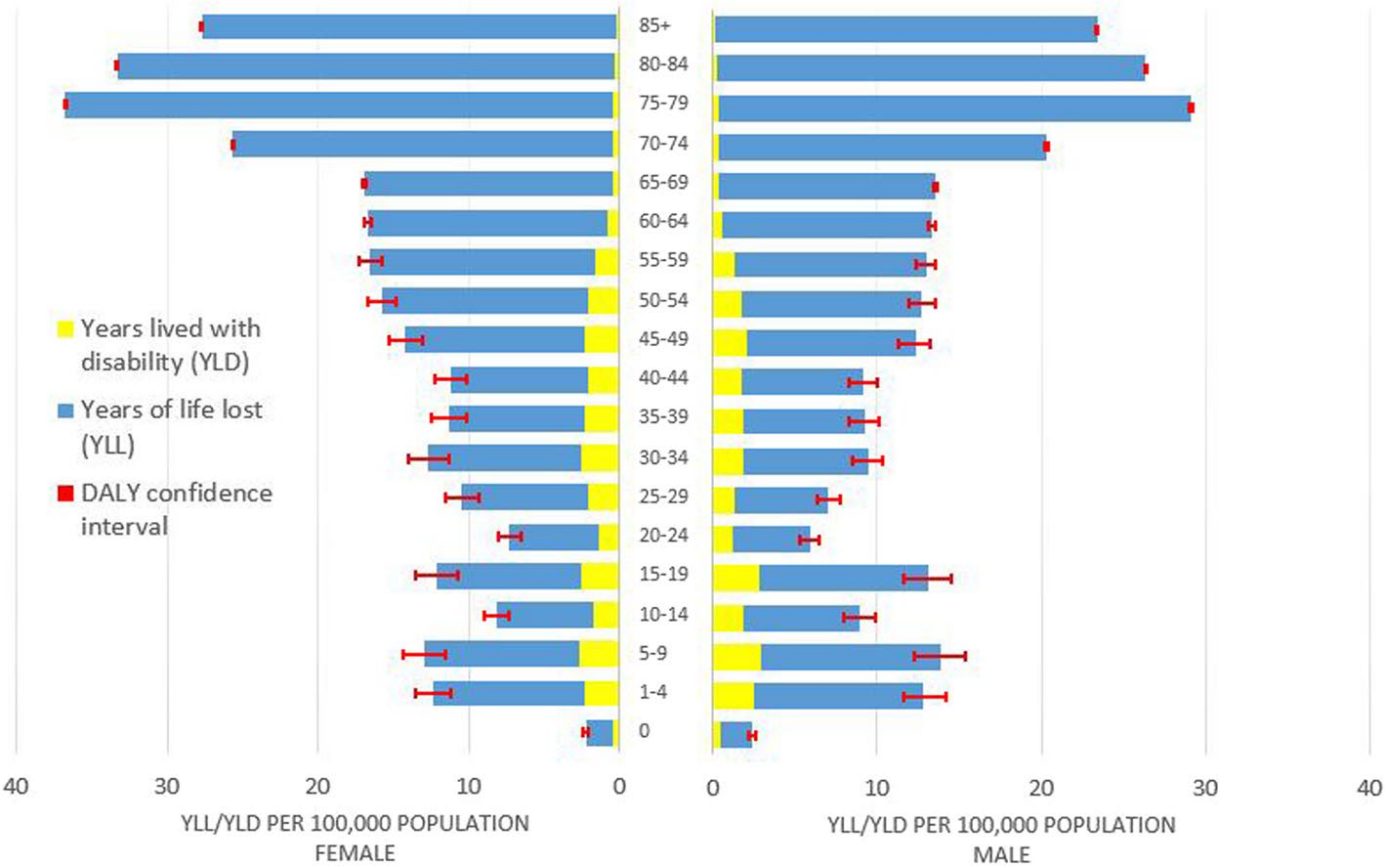
Disease burden baseline scenario, 2015

For all baseline, lower-bound and upper-bound scenarios, the main contributor to the total burden are sequelae after a symptomatic infection with an average between 9% and 99%. The remaining burden can be attributed to the disability caused by an acute infection (Fig. 3).

### Disease burden by specific health outcomes and sequelae

In baseline scenarios, pneumonia accounts for 56% of the total disease burden, followed by encephalitis (19%), myocarditis (12%), and chronic bronchitis (8%). Other sequelae contribute 6% of the total burden. In lower-bound scenarios, pneumonia (49%) and chronic bronchitis (17%) are the leading causes, followed by encephalitis (13%), myocarditis (10%), and others (11%). In upper-bound scenarios, pneumonia (34%) is the predominant cause, followed by ARDS (32%), encephalitis (12%), myocarditis (7%) and others (15%).

### Disease burden by age and sex

The highest overall burden is shared by the three oldest age groups (75 - 79; 80 - 84; 85+), while the lowest burden is found in age groups 15 - 19, 20 - 24, and 25 - 29. Burden generally increases with age from the age group 30 - 34 and older. Infants under one year and children aged 1 - 4 years have comparable burden estimates to age groups 40 - 44 and 45 - 49. The YLL is the main contributor to the total burden across all ages and scenarios for both males and females. YLD shares remain relatively constant until the age group 60 - 64, after which YLL becomes the predominant cause of burden for older age groups. Age and burden trends are similar for both sexes, with higher total burden observed in older age groups for females compared to males (Fig. 3).

## Discussion

The disease burden estimates presented in this study provide first results for the national seasonal influenza burden for the period between 2015 and 2020 in Germany. Using a composite health measure that considers both mortality and morbidity, the study provides valuable insights into the impact of the disease on population health. Three different scenarios were developed to calculate a reasonable range of possible DALY estimates. Compared to previous efforts, this study reports substantially higher DALY estimates for all scenarios, ranging from 127,100 DALYs (153 DALYs per 100,000 population) to 1,171,115 DALYs (1,414 DALYs per 100,000 population) (Tables 2 and 3). Various factors are contributing to this effect. Previous exercises either did not correct for underestimation or relied on GP consultation rates to estimate multiplication factors. This plays an important role in explaining the higher estimates in the current exercise, as multiplication factors used in the present study range from 7 to 121, depending on age and sex. Previous exercises either used MFs ranging from 4.12 to 5.12 [5], used a GP consultation rate of 30% [4], used a symptomatic attack rate of 1 to 2% [8] in the population, or did not perform any adjustments [1, 3], yielding lower YLL, YLD, and DALY estimates. Thus, influenza-related consultation numbers presented by the AGI were considered appropriate to estimate the true number of yearly symptomatic influenza cases and multiplication factors. However, due to limited information on age-specific consultation, surveillance data reporting incidences for each year of age up to 80 were used in combination with multiplication factors. Influenza-related consultations derived from the AGI ranged between 3.6 million and 9 million, in line with yearly attack rates between 5% and 20% reported by the RKI, resulting in a 3 - 6-fold increase in assumed symptomatic influenza cases and 4 - 35-fold increase in the disease burden compared to the modeling approach presented by Plass et al. [8, 63] (Table 2). Additional health outcomes and sequelae were considered in the model presented, with myocarditis, encephalitis, sinusitis, bronchitis, and chronic bronchitis considered as possible consequences for the first time in measuring the disease burden of seasonal influenza. Following the addition of possible sequelae, higher disease burden estimates were identified. In line with calculations reported by Cassini et al., the main contributor to the total disease burden is premature death (YLL). Considering available evidence, previous publications assumed a fatality rate of 0.1%, including both complicated and uncomplicated influenza cases. This estimate was derived from findings in the “Community Strategy for Pandemic Influenza Mitigation in the United States” from 2007, preceding the influenza-H1N1 pandemic [64]. Death after symptomatic infection was therefore the only instance of premature death in previous modeling approaches, with all other sequelae contributing to YLD only, potentially leading to overestimation of YLDs. Further analyses applying the previous modeling approach resulted in considerably lower DALY estimates for all scenarios (see Additional file 3). Using a single category of death encompassing both complicated and uncomplicated, presuming a fatality rate of 0.1%, could lead to an underestimation of the overall disease burden. Introducing a higher fatality rate might provide a more accurate estimation of the overall burden, however, information on relative contribution of possible sequelae is lost. In their weekly influenza surveillance report, the Centers for Disease Control and Prevention provide mortality estimates due to influenza for seasons 2019-20 through 2022-23, ranging from 0.01% to 1,5% [65]. The estimates presented in this study give a good approximation of each sequela’s contribution to the overall disease burden. In all scenarios presented, pneumonia, acute infection, encephalitis, myocarditis, and chronic bronchitis account for over 90% of the total disease burden. While other sequelae combined only amount to 10% of the total disease burden, these sequelae constitute most previous outcome trees used for calculating disease burden estimates. This underlines the importance of including sequelae beyond respiratory illnesses. Stratifying the disease burden by age and sex, a higher disease burden is observed in infants, younger age groups, and in the oldest age groups. Accordingly, the highest incidences of seasonal influenza reported by the RKI in Germany lie within the same age groups, underlining the importance of vaccination coverage. In 2021, the Netherlands, Ireland and Denmark reported vaccination rates of over 70% in people aged 65 years and over, while Germany reported a rate of 47.3%, declining by 8.8% since 2011. These differences can significantly impact the observed complication rates of symptomatic influenza cases.

### Limitations

Important limitations should be noted when interpreting the results. The clinical case-definition for influenza changed in 2019, introducing new definitions (D and E) in addition to the existing ones (B and C). The new definition is more sensitive and likely led to an increase in notified cases, including asymptomatic ones [66]. This change affects the comparability of notified cases before and after 2019, and should be considered when comparing DALY trends to consultation numbers and incidences. Transition probabilities for death, ARDS, myocarditis, encephalitis, and pneumonia may be overestimated due to the inclusion of publications from different regions and limited country-specific data on influenza-related complications in Germany. Studies primarily focused on inpatient settings, potentially leading to further overestimation. Age-specific transition probabilities had limited information, resulting in age redistribution only applied to pneumonia, death due to pneumonia, and ARDS. Assuming a higher death rate for most complications at very young and higher age, the disease burden in those age groups could have been underestimated. Disability weights were derived from the most recent GBD publication or previous models, with proxy disability weights used when data was lacking (e.g., COPD disability weights for ARDS). In the absence of reported 2020 influenza-related consultations, the multiplication factors for 2019 were used as a proxy. Due to the number of reported cases and incidences being almost identical for 2019 and 2020, similar number of consultations was assumed for both years. Age group 80 and older data was used as an approximation for the age group 85 and older, potentially underestimating disease burden in the latter group due to increasing mortality with age. Death counts by cause vary due to improper reporting and documentation, particularly for influenza. According to the RKI, it is common not to attribute the cause of death to influenza after clinical evaluations are performed. The cause of death is usually attributed to other conditions, even in the presence of laboratory confirmed influenza. This is due to the fact that it is difficult to determine the exact cause of death and whether a death occurred with influenza or rather due to it, considering all comorbidities. In the 2018/2019 Influenza Epidemiology Report in Germany, conservative excess deaths from influenza were estimated and were 15 to 78 times higher than confirmed influenza deaths. The present study reports death counts 0.4 to 4 times higher than excess deaths reported by the Robert Koch-Institute, aligning with previous conservative estimates [19]. Hospital discharge data in Germany shows an average of 19,300 sepsis deaths per year, while this study estimates an average of 115 influenza-related sepsis deaths annually. Similar patterns are observed for myocarditis, with 17,400 hospital deaths and 433 to 1,084 influenza-related deaths in this study. However, higher estimates are found for ARDS and encephalitis in hospital discharge data (950 deaths and 170 deaths per year, respectively) compared to the study’s estimates (498 to 10,882 ARDS deaths and 433 to 1,084 encephalitis deaths due to influenza), indicating possible overestimation of transition probabilities [67]. Depending on the year under investigation, estimates on DALYs per 100.000 population for the baseline and lower-bound scenario are comparable to the overall disease burden caused by lower respiratory infections in Germany (374 per 100.000 population) and other western European countries (Netherlands: 407 per 100.000 population; France: 354 per 100.000 population; Sweden: 302 per 100.000 population) according to the Global Burden of Disease Study 2019. However, results from upper-bound scenarios equate to the burden of chronic diseases such as Diabetes Type 2, lower back pain and ischemic heart disease in Germany, indicating an overestimation. Compared to other infectious diseases, the total burden is comparable to that caused by lower respiratory infections in Western European countries. In contrast, Tuberculosis, HIV/AIDS, Acute Hepatitis B and Measles are cause for considerably lower total DALYs in Western, Central, Eastern European and North American countries. Estimates per 100,000 population are similar to those for Tuberculosis in Lithuania and Ukraine, HIV/AIDS in Estonia and Ukraine, and lower respiratory tract infections in most of these regions [66] (Table 1). Transition probabilities and disability weights were assumed to be constant over time, without considering changes resulting from intervention programs or variations in natural immunity. This assumption is crucial for the disease burden of specific influenza subtypes, as different subtypes may have varying complication rates. Due to the limited evidence on subtype-specific complications, a single influenza model independent of subtypes was developed. Multimorbidity, the presence of multiple medical conditions in one person, can overestimate YLDs in the DALY approach. Adjusting for multimorbidity is complex due to varying onset and duration of different conditions. McDonald et al. suggested an individual-based modeling approach, but empirical evidence and practical solutions are lacking [68]. Further research is necessary to address multimorbidity in incidence-based modeling approaches. Lastly, search for literature was restricted to one database. As rapid reviews aim to yield similar conclusions as systematic reviews in a more time-efficient manner, it is likely that the inclusion of additional databases would have contributed to a more comprehensive information synthesis.

## Conclusions

Disease burden models provide insights into the history, progression, and consequences of a disease using composite health measures. The quality of these models depends on available information on complications, probabilities, underestimation, and severity. Building upon findings of the present study, future research and surveillance are necessary to understand the complications following a symptomatic influenza infection. Reliable data on disease burden estimates are also of need to identify vulnerable groups. As resources are limited, reliable data on prioritisation and allocation of resources are important for an informed and evidence-based decision-making process. Evaluations of interventions can provide information on DALYs averted, cost-effectiveness, and total expenditure. Studies on the underestimation of influenza cases are crucial to accurately assess the disease’s impact on population health. Research across regions and cultures can help understand the effects of interventions or their lack thereof, e.g., vaccination rates in the population. Our results show high burden estimates in vulnerable populations such as infants, children under age of five and elderly population, indicating the need of further research and vaccination programs in members of risk groups and their close contacts. Finally, considering influenza complications beyond respiratory conditions is important for a comprehensive understanding of the disease burden.

## Supplementary Information

Supplementary Material 1. Supplementary Table 1: Studies included in Rapid Review; Supplementary Table 2: Calculation of Multiplication Factors; Supplementary Table 3: Population Data; Supplementary Table 4: Scenario Input Parameters. Supplementary Table 1 gives information on all studies considered for the rapid review with information on author, study period, setting, country, populatioon, age range, and available data. Supplementary Table 2 gives information on the calculation of Multiplication Factors used, presenting the Influenza-related consultations reported by the AGI and Cases reported by the RKI. Supplementary Table 3 gives information on the population data used for the modeling approach, such as average number of cases per year, underestimation, age distribution, and life expectancy. Supplementary Table 4 presents all input data for all scenarios and sequelae. Information on probabilities, disability weights, duration in years and the source are given.

Supplementary Material 2. Detailed results for all years and scenarios.

Supplementary Material 3. Detailed results for all years and scenarios, using the previous modeling approach.

## Data Availability

The datasets supporting the conclusions of this article are available in the Zenodo repository, reference number 10.5281/zenodo.7368150, https://doi.org/10.5281/zenodo.10053813.

## Abbreviations

AGI: Arbeitsgemeinschaft influenza
ARDS: Acute respiratory distress syndrome
BCoDE: Burden of communicable disease in Europe
DALY: Disability-adjusted life years
ECDC: European centre for disease prevention and control
EEA: European economic area
EU: European union
GBD: Global burden of disease
MF: Mutliplication factor
RKI: Robert Koch-institute
WHO: World health organization
YLD: Years lived with disability
YLL: Years of life lost due to premature death

## References

[1] Cassini A, Colzani E, Pini A, Mangen MJ, Plass D, McDonald SA, et al. Impact of infectious diseases on population health using incidence-based disability-adjusted life years (DALYs): results from the Burden of Communicable Diseases in Europe study, European Union and European Economic Area countries, 2009 to 2013. Euro Surveill. 2018;23(16). 10.2807/1560-7917.ES.2018.23.16.17-00454 PMC5915974 29692315

[2] Global Burden of Disease Study (GBD) 2020. 2020. https://vizhub.healthdata.org/gbd-results/. Accessed 01 Aug 2023.

[3] van Lier AE, Havelaar AH, Nanda A. The burden of infectious diseases in Europe: a pilot study. Euro Surveill. 2007;12(12):E3-4. 18076860 10.2807/esm.12.12.00751-en

[4] Wielders CC, van Lier AE, van ’t Klooster TM, van Gageldonk-Lafeber AB, van den Wijngaard CC, Haagsma JA, et al. The burden of 2009 pandemic influenza A(H1N1) in the Netherlands. Eur J Public Health. 2012;22(1):150–7. 21183472 10.1093/eurpub/ckq187

[5] van Lier A, McDonald SA, Bouwknegt M, EPI group, Kretzschmar ME, Havelaar AH, et al. Disease Burden of 32 Infectious Diseases in the Netherlands, 2007-2011. PLoS One. 2016;11(4):e0153106. 10.1371/journal.pone.0153106 PMC4838234 27097024

[6] McDonald SA, van Lier A, Plass D, Kretzschmar ME. The impact of demographic change on the estimated future burden of infectious diseases: examples from hepatitis B and seasonal influenza in the Netherlands. BMC Public Health. 2012;12:1046. 23217094 10.1186/1471-2458-12-1046 PMC3537516

[7] Survstat@RKI2.0. 2023. https://survstat.rki.de/Content/Query/Create.aspx. Accessed 20 Aug 2023.

[8] Plass D, Mangen MJJ, Kraemer A, Pinheiro P, Gilsdorf A, Krause G, et al. The disease burden of hepatitis B, influenza, measles and salmonellosis in Germany: first results of the burden of communicable diseases in Europe study. Epidemiol Infect. 2014;142(10):2024–35. 24480146 10.1017/S0950268813003312 PMC9151280

[9] Macias AE, McElhaney JE, Chaves SS, Nealon J, Nunes MC, Samson SI, et al. The disease burden of influenza beyond respiratory illness. Vaccine. 2021;39:A6–14. 33041103 10.1016/j.vaccine.2020.09.048 PMC7545338

[10] Higgins JPT, Thomas J, Chandler J, Cumpston M, Li T, Page MJ, et al. CochraneHandbook for Systematic Reviews of Interventions. Version 6.4. Cochrane; 2023. https://training.cochrane.org/handbook. Accessed 01 Aug.

[11] Murray CJ, Lopez AD, Harvard School of Public Health, World Health Organization, World Bank. The global burden of disease: a comprehensive assessment of mortality and disability from diseases, injuries, and risk factors in 1990 and projected to 2020. Boston: Published by the Harvard School of Public Health on behalf of the World Health Organization and the World Bank; Distributed by Harvard University Press; 1996. ISBN: 0965546608.

[12] Arias-Fernandez L, San-Roman Montero J, Gil-Prieto R, Walter S, Gil de Miguel A. Burden of pneumonia in patients with viral and bacterial coinfection in Spain during six consecutive influenza seasons, from 2009-10 to 2014-15. Vaccine. 2021;39(35):5002–6. 34304929 10.1016/j.vaccine.2021.07.035

[13] Colzani E, Cassini A, Lewandowski D, Mangen MJ, Plass D, McDonald SA, et al. A Software Tool for Estimation of Burden of Infectious Diseases in Europe Using Incidence-Based Disability Adjusted Life Years. PLoS One. 2017;12(1):e0170662. 28107447 10.1371/journal.pone.0170662 PMC5249178

[14] Mangen MJ, Plass D, Havelaar AH, Gibbons CL, Cassini A, Muhlberger N, et al. The pathogen-and incidence-based DALY approach: an appropriate [corrected] methodology for estimating the burden of infectious diseases. PloS One. 2013;8(11):e79740. 10.1371/journal.pone.0079740 PMC3835936 24278167

[15] Robert Koch Institut. Bericht zur Epidemiologie der Influenza in Deutschland, Saison 2014/15. Berlin: Robert Koch Institut; 2015. ISBN: 978-3-89606-265-9. Available from: https://influenza.rki.de/Saisonbericht.aspx. Accessed 20 Aug 2023.

[16] Robert Koch Institut. Bericht zur Epidemiologie der Influenza in Deutschland, Saison 2015/16. Berlin: Robert Koch Institut; 2016. ISBN: 978-3-89606-275-8. 10.17886/rkipubl-2016-010. Available from: https://influenza.rki.de/Saisonbericht.aspx. Accessed 20 Aug 2023.

[17] Robert Koch Institut. Bericht zur Epidemiologie der Influenza in Deutschland, Saison 2016/17. Berlin: Robert Koch Institut; 2017. ISBN: 978-3-89606-290-1. 10.17886/rkipubl-2017-009. Available from: https://influenza.rki.de/Saisonbericht.aspx. Accessed 20 Aug 2023.

[18] Robert Koch Institut. Bericht zur Epidemiologie der Influenza in Deutschland, Saison 2017/18. Berlin: Robert Koch Institut; 2018. ISBN: 978-3-89606-293-2. 10.17886/rkipubl-2018-003. Available from: https://influenza.rki.de/Saisonbericht.aspx. Accessed 20 Aug 2023.

[19] Robert Koch Institut. Bericht zur Epidemiologie der Influenza in Deutschland, Saison 2018/19. Berlin: Robert Koch Institut; 2019. ISBN: 978-3-89606-301-4. 10.25646/6232. Available from: https://influenza.rki.de/Saisonbericht.aspx. Accessed 20 Aug 2023.

[20] An der Heiden M, Buchholz U, Buda S. Estimation of influenza- and respiratory syncytial virus-attributable medically attended acute respiratory infections in Germany, 2010/11-2017/18. Influenza Other Respir Viruses. 2019;13(5):517–21. 31339223 10.1111/irv.12666 PMC6692544

[21] World Health Organization. WHO methods and data sources for global burden of disease estimates 2000-2019. 2020. WHO/DDI/DNA/GHE/2020.3. https://cdn.who.int/media/docs/default-source/gho-documents/global-health-estimates/ghe2019_daly-methods.pdf. Accessed 01 Aug 2023.

[22] GENESIS-Online. 2023. https://www-genesis.destatis.de/genesis/online. Accessed 01 Aug 2023.

[23] Podewils LJ, Liedtke LA, McDonald LC, Hageman JC, Strausbaugh L, Fischer TK, et al. A national survey of severe influenza-associated complications among children and adults, 2003–2004. Clin Infect Dis. 2005;40(11):1693–6. 15889371 10.1086/430424

[24] Bhat N, Wright JG, Broder KR, Murray EL, Greenberg ME, Glover MJ, et al. Influenza-associated deaths among children in the United States, 2003–2004. N Engl J Med. 2005;353(24):2559–67. 16354892 10.1056/NEJMoa051721

[25] Piroth L, Cottenet J, Mariet AS, Bonniaud P, Blot M, Tubert-Bitter P, et al. Comparison of the characteristics, morbidity, and mortality of COVID-19 and seasonal influenza: a nationwide, population-based retrospective cohort study. Lancet Respir Med. 2021;9(3):251–9. 33341155 10.1016/S2213-2600(20)30527-0 PMC7832247

[26] Marano G, Pariani E, Luconi E, Pellegrinelli L, Galli C, Magoni M, et al. Elderly people: propensity to be vaccinated for seasonal influenza in Italy. Hum Vaccines Immunotherapeutics. 2020;16(8):1772–81. 10.1080/21645515.2019.1706931 PMC7482847 32040352

[27] Meier CR, Napalkov PN, Wegmuller Y, Jefferson T, Jick H. Population-based study on incidence, risk factors, clinical complications and drug utilisation associated with influenza in the United Kingdom. Eur J Clin Microbiol Infect Dis. 2000;19(11):834–42. 11152308 10.1007/s100960000376

[28] Oliveira EC, Marik PE, Colice G. Influenza pneumonia: a descriptive study. Chest. 2001;119(6):1717–23. 11399696 10.1378/chest.119.6.1717

[29] Sessa A, Costa B, Bamfi F, Bettoncelli G, D’Ambrosio G. The incidence, natural history and associated outcomes of influenza-like illness and clinical influenza in Italy. Fam Pract. 2001;18(6):629–34. 11739352 10.1093/fampra/18.6.629

[30] Kaiser L, Wat C, Mills T, Mahoney P, Ward P, Hayden F. Impact of oseltamivir treatment on influenza-related lower respiratory tract complications and hospitalizations. Arch Intern Med. 2003;163(14):1667–72. 12885681 10.1001/archinte.163.14.1667

[31] Peltola V, Ziegler T, Ruuskanen O. Influenza A and B virus infections in children. Clin Infect Dis. 2003;36(3):299–305. 12539071 10.1086/345909

[32] Heikkinen T, Silvennoinen H, Peltola V, Ziegler T, Vainionpaa R, Vuorinen T, et al. Burden of influenza in children in the community. J Infect Dis. 2004;190(8):1369–73. 15378427 10.1086/424527

[33] Moore DL, Vaudry W, Scheifele DW, Halperin SA, Dery P, Ford-Jones E, et al. Surveillance for influenza admissions among children hospitalized in Canadian immunization monitoring program active centers, 2003–2004. Pediatrics. 2006;118(3):e610-9. 16950953 10.1542/peds.2005-2744

[34] Barr CE, Schulman K, Iacuzio D, Bradley JS. Effect of oseltamivir on the risk of pneumonia and use of health care services in children with clinically diagnosed influenza. Curr Med Res Opin. 2007;23(3):523–31. 17355734 10.1185/030079906x167499

[35] Coffin SE, Zaoutis TE, Rosenquist AB, Heydon K, Herrera G, Bridges CB, et al. Incidence, complications, and risk factors for prolonged stay in children hospitalized with community-acquired influenza. Pediatrics. 2007;119(4):740–8. 17403845 10.1542/peds.2006-2679

[36] Kwong KL et al. Influenza-related hospitalisations in children. J Paediatr Child Health. 2009;45(11):660–4. 19845841 10.1111/j.1440-1754.2009.01591.x

[37] Lee N, Choi KW et al. Outcomes of adults hospitalised with severe influenza. Thorax. 2010;65(6):510–5. 20522848 10.1136/thx.2009.130799

[38] Belongia EA et al. Clinical characteristics and 30-day outcomes for influenza A 2009 (H1N1), 2008–2009 (H1N1), and 2007–2008 (H3N2) infections. JAMA. 2010;304(10):1091–8. 20823435 10.1001/jama.2010.1277

[39] Bassetti M et al. Risk factors for severe complications of the novel influenza A (H1N1): analysis of patients hospitalized in Italy. Clin Microbiol Infect. 2011;17(2):247–50. 20518797 10.1111/j.1469-0691.2010.03275.x

[40] Lee N, Chasn PKS et al. Complications and outcomes of pandemic 2009 Influenza A (H1N1) virus infection in hospitalized adults: how do they differ from those in seasonal influenza? J Infect Dis. 2011;203(12):1739–47. 21606532 10.1093/infdis/jir187

[41] Viasus D et al. Pneumonia complicating pandemic (H1N1) 2009: risk factors, clinical features, and outcomes. Medicine (Baltimore). 2011;90(5):328–36. 21862936 10.1097/MD.0b013e31822e67a7

[42] Mansour MM et al. 2009 H1N1 influenza A in children: a descriptive clinical study. Ann Saudi Med. 2012;32(1):59–63. 22156647 10.5144/0256-4947.2012.59 PMC6087646

[43] Jain S et al. Pandemic Influenza AVHIT. Influenza-associated pneumonia among hospitalized patients with 2009 pandemic influenza A (H1N1) virus–United States, 2009. Clin Infect Dis. 2012;54(9):1221–1229. 10.1093/cid/cis197 22437239

[44] Wieching A et al. Clinical characteristics of pediatric hospitalizations associated with 2009 pandemic influenza A (H1N1) in Northern Bavaria, Germany. BMC Res Notes. 2012;5:304. 22713762 10.1186/1756-0500-5-304 PMC3438061

[45] Lee CY et al. Epidemiology, clinical features, treatment, and outcomes of cases of influenza A infection during the 2009 influenza pandemic in northern Taiwan. Pediatr Neonatol. 2012;53(4):257–63. 22964284 10.1016/j.pedneo.2012.06.005

[46] Hernandez-Bou S et al. SSoPE IDWG. Hospitalized children with influenza A H1N1 (2009) infection: a Spanish multicenter study. Pediatr Emerg Care. 2013;29(1):49–52. 10.1097/PEC.0b013e31827b528f 23283263

[47] Esterman EE et al. Influenza infection in infants aged 6 months during the H1N1-09 pandemic: a hospital-based case series. J Paediatr Child Health. 2013;49(8):635–40. 10.1111/jpc.12266 23782402

[48] Dawood FS et al. Complications and associated bacterial coinfections among children hospitalized with seasonal or pandemic influenza, United States, 2003–2010. J Infect Dis. 2014;209(5):686–94. 23986545 10.1093/infdis/jit473

[49] Chio SC et al. Molecular epidemiologic and clinical characteristics of influenza B-associated complications among hospitalized patients during an outbreak in Taiwan. Int J Infect Dis. 2014;23:94–100. 24721164 10.1016/j.ijid.2014.02.017

[50] Reed C et al. Complications among adults hospitalized with influenza: a comparison of seasonal influenza and the 2009 H1N1 pandemic. Clin Infect Dis. 2014;59(2):166–74. 24785230 10.1093/cid/ciu285 PMC7314251

[51] Hagerman A et al. Group PID. Clinical characteristics and outcomes in children hospitalised with pandemic influenza A/H1N1/09 virus infection-a nationwide survey by the Pediatric Infectious Diseases Group of Switzerland. Swiss Medical Weekly. 2015;145(3132):w14171. 10.4414/smw.2015.14171 26217892

[52] Shah NS et al. Severe Influenza in 33 US Hospitals, 2013–2014: Complications and Risk Factors for Death in 507 Patients. Infect Control Hosp Epidemiol. 2015;36(11):1251–60. 26224364 10.1017/ice.2015.170

[53] Puig-Barbera J et al. Epidemiology of Hospital Admissions with Influenza during the 2013/2014 Northern Hemisphere Influenza Season: Results from the Global Influenza Hospital Surveillance Network. PloS one. 2016;11(5):e0154970. 27196667 10.1371/journal.pone.0154970 PMC4873033

[54] Vardakas KZ et al. Impact of oseltamivir use on the reduction of complications in patients with influenza: a prospective study. Arch Virol. 2016;161(9):2511–8. 27368992 10.1007/s00705-016-2941-5

[55] Zanuzdana et al. Influenza and community acquired pneumonia in German primary care. Bundesgesundheitsbl Gesundheitsforsch Gesundheitsschutz. 2016;59(11):1492–502. 10.1007/s00103-016-2442-4 27695937

[56] Topoulos S et al. Analysis of acute respiratory infections due to influenza virus A, B and RSV during an influenza epidemic 2018. Infection. 2019;47(3):425–33. 30649684 10.1007/s15010-018-1262-x

[57] Chong CY et al. The burden and clinical manifestation of hospitalized influenza among different pediatric age-groups in the tropics. Influenza Other Respir Viruses. 2020;14(1):46–54. 31608598 10.1111/irv.12692 PMC6928028

[58] Teutsch SM et al. Ten Years of National Seasonal Surveillance for Severe Complications of Influenza in Australian Children. Pediatr Infect Dis J. 2021;40(3):191–8. 33093432 10.1097/INF.0000000000002961

[59] Sharma Y et al. Clinical characteristics and outcomes of influenza A and B virus infection in adult Australian hospitalised patients. BMC Infect Dis. 2020;20(1):913. 33261559 10.1186/s12879-020-05670-8 PMC7705848

[60] Hjalmarsson A, Blomqvist P, Brytting M, Linde A, Skoldenberg B. Encephalitis after influenza in Sweden 1987–1998: a rare complication of a common infection. Eur Neurol. 2009;61(5):289–94. 19295216 10.1159/000206854

[61] Wilking AN, Elliott E, Garcia MN, Murray KO, Munoz FM. Central nervous system manifestations in pediatric patients with influenza A H1N1 infection during the 2009 pandemic. Pediatr Neurol. 2014;51(3):370–6. 25160541 10.1016/j.pediatrneurol.2014.04.026

[62] Mylonaki E et al. Neurological complications associated with influenza in season 2017/18 in Austria-a retrospective single center study. J Clin Virol. 2020;127:104340. 32302952 10.1016/j.jcv.2020.104340

[63] Robert Koch-Institut. RKI-Ratgeber: Influenza. 2018. https://www.rki.de/DE/Content/Infekt/EpidBull/Merkblaetter/Ratgeber_Influenza_saisonal.html. Accessed 20 Aug 2023.

[64] Centers for Disease Control and Prevention (U.S.). Interim pre pandemic planning guidance: community strategy for pandemic influenza mitigation in the United States: early, targeted, layered use of nonpharmaceutical interventions. Atlanta: Centers for Disease Control and Prevention; 2007. Available from: https://stacks.cdc.gov/view/cdc/11425. Accessed 22 Aug 2023.

[65] Weekly U.S. Influenza Surveillance Report 2023. 2023. https://www.cdc.gov/flu/weekly/index.htm. Accessed 07 Aug 2023.

[66] Robert Koch Institut. Falldefinitionen des Robert Koch Instituts zur Übermittlung von Erkrankungs oder Todesfällen und Nachweisen von Krankheitserregern. Ausgabe. Berlin: Robert Koch Institut; 2019. ISSN: 2363 7897. Available from: https://www.rki.de/DE/Themen/Infektionskrankheiten/Meldewesen/Falldefinitionen/Archiv/Falldefinitionen_des_RKI_2019.pdf?__blob=publicationFile&v=1. Accessed 20 Aug 2023. 10.1007/s00103-016-2355-2 27221548

[67] GENESIS-Online. 2023. https://www.destatis.de/DE/Themen/Gesellschaft-Umwelt/Gesundheit/Krankenhaeuser/_inhalt.html#sprg475696. Accessed 01 Aug 2023.

[68] McDonald SA, Haagsma JA, Cassini A, Devleesschauwer B. Adjusting for comorbidity in incidence-based DALY calculations: an individual-based modeling approach. BMC Med Res Methodol. 2020;20(1):100. 32375653 10.1186/s12874-020-00987-z PMC7201540

