# Supplementary material for: Measuring the disease burden of seasonal influenza in Germany 2015 - 2020 using the incidence-based disability-adjusted life years (DALYs)
Source: BMC Infect Dis. 2025 Mar 26;25:413. doi: 10.1186/s12879-025-10613-2 (PMC11948870; doi:10.1186/s12879-025-10613-2)
Supplement: Supplementary file 1 — Supplementary Material 1. Supplementary Table 1: Studies included in Rapid Review; Supplementary Table 2: Calculation of Multiplication Factors; Supplementary Table 3: Population Data; Supplementary Table 4: Scenario Input Parameters. Supplementary Table 1 gives information on all studies considered for the rapid review with information on author, study period, setting, country, populatioon, age range, and available data. Supplementary Table 2 gives information on the calculation of Multiplication Factors used, presenting the Influenza-related consultations reported by the AGI and Cases reported by the RKI. Supplementary Table 3 gives information on the population data used for the modeling approach, such as average number of cases per year, underestimation, age distribution, and life expectancy. Supplementary Table 4 presents all input data for all scenarios and sequelae. Information on probabilities, disability weights, duration in years and the source are given. [file 12879_2025_10613_MOESM1_ESM.pdf]

Supplementary Table 1: Studies included in Rapid Review

Author	Study Period	Setting	Country	N (pop.)	Age range	Available data (Probabilities)
Meier et al. (2000) (27)	1991 – 1996	Outpatient	UK	141.293	All ages	Pneumonia (0.38%), Otitis media (1.05%), Bronchitis (1.48%), Myocarditis (0.08%), Sinusitis (0.03%), Death (0.19%)
Oliveira et al. (2001) (28)	1999 – 2000	Inpatient	USA	35	25 – 92 Years	Pneumonia (48.5%), Death (29.4%), Pneumonia Recovery (80.5%)
Sessa et al. (2001) (29)	1998 – 1999	Outpatient	Italy	6192	All ages	Pneumonia (1.4%), Otitis media (1.6%), Bronchitis (14.7%), Sinusitis (3.2%)
Kaiser et al. (2003) (30)	1997 – 2000	Outpatient	USA, Northern Hemisphere, Southern Hemisphere	3564	13 – 97 Years	Pneumonia (1.8%), Bronchitis (8.2%)
Peltola et al. (2003) (31)	1980 – 1999	Inpatient	Finland	683	<18 Years	Pneumonia (9%), Otitis media (24%), Encephalitis (0.7%)
Heikkinen et al. (2004) (32)	2000 – 2002	Outpatient	Finland	372	<13 Years	Pneumonia (2.4%), Otitis media (23%), Sinusitis (3.5%)
Podewils et al (2005) (23) (cross-sectional)	2003 – 2004	Inpatient / Outpatient	USA	13.560	N/A	Death (0.4%)
Bhat et al. (2005) (24) (cross-sectional)	2003 – 2004	Inpatient / Outpatient	USA	153	<18 Years	Death (0.00021%)
Moore et al. (2006) (33)	2003 – 2004	Inpatient	Canada	505	N/A	Pneumonia (6.3%), Otitis media (3%), Myocarditis (0.4%), Sinusitis (0.4%), Death (0.6%)
Barr et al. (2007) (34)	2000 – 2004	Inpatient / Outpatient	USA	24.854	1 – 12 Years	Pneumonia (1.2%)
Coffin et al. (2007) (35)	2000 – 2004	Inpatient	USA	745	<21 Years	Pneumonia (15%), Death (1%)

Hjalmarsson et al. (2009) (36)	1987 – 1998	Inpatient	Sweden	14.250	All Ages	Encephalitis (0.07%), Death (4.1%)
Kwong et al. (2009) (37) (cross sectional)	2005	Inpatient	Hong-Kong	123	>18 Years	Pneumonia (13%), Otitis media (0.8%), Bronchitis (4.1%), Encephalitis (0.8%), Sinusitis (0.8%)
Lee et al. (2010) (38)	2007 – 2008	Inpatient	Hong-Kong	754	<18 Years	Pneumonia (43%), Bronchitis (5.4%), Death (5.2%)
Belongia, E.A. (2010) (39)	2007 – 2009	Inpatient / Outpatient	USA	1398	All Ages	Pneumonia (2%), Otitis media (9.5%), Sinusitis (6.6%)
Bassetti et al. (2011) (40)	2009	Inpatient	Italy	81	All Ages	Pneumonia (50%), Death (3%)
Lee et al. (2011) (41)	2007 – 2010	Inpatient	Hong-Kong	382	>16 Years	Pneumonia (34.3%), Death (3.1%)
Viasus et al. (2011) (42)	2009	Inpatient	Spain	542	>18 Years	Pneumonia (43%)
Mansour, M.M.H.K. & Al-Hadidi, K.H.A. (2012) (43)	2009 – 2010	Inpatient	Saudi-Arabia	89	<15 Years	Pneumonia (31.5%), Bronchitis (33.7%)
Jain et al. (2012) (44) (Case-Series)	2009	Inpatient	USA	451	All Ages	Pneumonia (43%), ARDS (6.2%), Sepsis (4.6%), Death (8.2%)
Wieching et al. (2012) (45)	2009 – 2010	Inpatient	Germany	94	<18 Years	Pneumonia (17%), Otitis media (3%), Bronchitis (21%), ARDS (1%)
Lee et al. (2012) (46)	2009	Inpatient / Outpatient	Taiwan	1158	All Ages	Pneumonia (1.9%), Otitis media (0.3%), Bronchitis (0.3%), Encephalitis (0.1%), Sinusitis (0.3%)
Hernández-Bou et al. (2013) (47) (Case-Series)	2009	Inpatient	Spain	308	<14 Years	Pneumonia (44.8%), Encephalitis (1.3%), Myocarditis (0.6%), Sepsis (3.9%), Death (1.9%)

Esterman et al. (2013) (48)	2009	Inpatient	Australia	32	<6 Months	Pneumonia (22%), Encephalitis (1%)
Dawood et al. (2014) (49)	2003 – 2010	Inpatient	USA	6769	<18 Years	Pneumonia (36%), Otitis media (6%), Bronchitis (0.17%), Myocarditis (0.08%), Sepsis (3%), Sinusitis (0.25%), Death (1%)
Chiu et al. (2014) (50)	2011 – 2012	Inpatient	Taiwan	326	All Ages	Pneumonia (54%), Encephalitis (2.1%), ARDS (49.4%), Death (26.6%)
Reed et al. (2014) (51)	2005 – 2010	Inpatient	USA	10.232	>18 Years	Pneumonia (39%), Myocarditis (0.3%), Sepsis (7.8%), Death (3.8%)
Wilking et al. (2014) (52)	2009 – 2010	Inpatient	USA	365	<13 Years	Encephalitis (1.1%)
Hagerman et al. (2015) (53)	2009	Inpatient	Switzerland	326	<18 Years	Pneumonia (25.8%), Otitis media (8.3%), Death (0.6%)
Shah et al. (2015) (54)	2013 – 2014	Inpatient	USA	507	All Ages	Pneumonia (31%), ARDS (39.6%), Death (19.1%)
Puig-Barberá et al. (2016) (55)	2013 – 2014	Inpatient	Russia, Turkey, China, Spain	5303	All Ages	Pneumonia (17.9%), ARDS (0.9%)
Vardakas et al. (2016) (56)	2011 – 2012	Outpatient	Greece	410	All Ages	Pneumonia (2%), Otitis media (0.6%), Bronchitis (7.4%), Sinusitis (2.2%)
Zanuzdana et al. (2016) (57)	2012 – 2015	Outpatient	Germany	156.803	All Ages	Pneumonia (1.6%)
Topolous et al. (2019) (58)	2018	Inpatient	Germany	212	All Ages	Pneumonia (24.2%), Death (4.7%), Pneumonia Recovery (84%)
Chong et al. (2020) (59)	2013 – 2014	Inpatient	Singapore	1272	<19 Years	Pneumonia (4.4%), Otitis media (0.63%), Bronchitis (0.94%), Encephalitis (0.47%), ARDS (0.47%), Sinusitis

						(0.24%), Death (0.2%)
Mylonaki et al. (2020) (60)	2017 – 2018	Inpatient	Austria	874	>18 Years	Encephalitis (0.22%)
Teutsch et al. (2021) (61)	2008 – 2017	Inpatient	Australia	613	<15 Years	Pneumonia (54.3%), Myocarditis (2.4%), Death (4.9%)
Sharma et al. (2020) (62)	2016 – 2020	Inpatient	Australia	1846	<18 Years	Pneumonia (7.2%)
Piroth et al. (2021) (63)	2018 – 2019	Inpatient	France	45.189	All Ages	Death (5.8%)
Marano et al. (2021) (26)	2014 – 2017	Inpatient / Outpatient	Italy	953.193	>65 Years	Death (1.5%)
Arias-Fernández et al. (2021) (12)	2009 – 2015	Inpatient	Spain	1993	>60 Years	Pneumonia Recovery (83.6%)

Formula for population size weighting:

$$\sum \frac{n_{ix}}{N_{ix} \times p_x}$$

n_{ix} = Sample size of study i investigating complication x

N_{ix} = Total sample size of studies i investigating complication x

P_x = Probability of complication x

Supplementary Table 2: Calculation of Multiplication Factors

	**Influenza-related consultations reported by AGI**	**Cases reported by RKI**	**Multiplication Factor**
2014/2015	0 – 4: 370,000<br>5 – 14: 680,000<br>15 – 34: 1,500,000<br>35 – 59: 2,700,000<br>60+: 900,000	0 – 4: 10,497<br>5 – 14: 13,416<br>15 – 34: 12,374<br>35 – 59: 25,454<br>60+: 15,816	0 – 4: 35<br>5 – 14: 51<br>15 – 34: 121<br>35 – 59: 106<br>60+: 57

2015/2016	0 – 4: 400,000<br>5 – 14: 700,000<br>15 – 34: 1,100,000<br>35 – 59: 1,400,000<br>60+: 400,000	0 – 4: 11,545<br>5 – 14: 16,430<br>15 – 34: 13,300<br>35 – 59: 16,361<br>60+: 7123	0 – 4: 35<br>5 – 14: 43<br>15 – 34: 83<br>35 – 59: 86<br>60+: 56
2016/2017	0 – 4: 600,000<br>5 – 14: 700,000<br>15 – 34: 1,300,000<br>35 – 59: 2,400,000<br>60+: 900,000	0 – 4: 8853<br>5 – 14: 13,151<br>15 – 34: 15,938<br>35 – 59: 28,967<br>60+: 28,936	0 – 4: 68<br>5 – 14: 53<br>15 – 34: 82<br>35 – 59: 83<br>60+: 31
2017/2018	0 – 4: 800,000<br>5 – 14: 1,100,000<br>15 – 34: 2,000,000<br>35 – 59: 3,900,000<br>60+: 1,300,000	0 – 4: 26,632<br>5 – 14: 36,267<br>15 – 34: 39,342<br>35 – 59: 97,510<br>60+: 68,933	0 – 4: 30<br>5 – 14: 30<br>15 – 34: 50<br>35 – 59: 40<br>60+: 19
2018/2019	0 – 4: 440,000<br>5 – 14: 500,000<br>15 – 34: 900,000<br>35 – 59: 1,700,000<br>60+: 350,000	0 – 4: 26,602<br>5 – 14: 22,567<br>15 – 34: 34,941<br>35 – 59: 62,017<br>60+: 47,045	0 – 4: 17<br>5 – 14: 26<br>15 – 34: 22<br>35 – 59: 27<br>60+: 7
2019/2020*<br><br>*Data not available, Mult. Factors assumed to be identical to season 2018/2019	-	-	0 – 4: 17<br>5 – 14: 26<br>15 – 34: 22<br>35 – 59: 27<br>60+: 7

Supplementary Table 3: Population Data

	**2015**		**2016**		**2017**	
Average number of cases per year	Female	Male	Female	Male	Female	Male
	0: 536	0: 700	0: 595	0: 787	0: 594	0: 618
	1 – 4: 4,305	1 – 4: 4,914	1 – 4: 4,749	1 – 4: 5,377	1 – 4: 3,649	1 – 4: 3,972
	5 – 9: 3,703	5 – 9: 4,306	5 – 9: 5,592	5 – 9: 6,287	5 – 9: 3.241	5 – 9: 3,688
	10 – 14: 2,399	10 – 14: 2,913	10 – 14: 2,528	10 – 14: 2,879	10 – 14: 2,885	10 – 14: 3,315
	15 – 19: 1,716	15 – 19: 2,019	15 – 19: 1,677	15 – 19: 2,026	15 – 19: 2,626	15 – 19: 2,702
	20 – 24: 1,026	20 – 24: 893	20 – 24: 1,061	20 – 24: 940	20 – 24: 1,624	20 – 24: 1,298
	25 – 29: 1,598	25 – 29: 1,122	25 – 29: 1,949	25 – 29: 1,513	25 – 29: 1,937	25 – 29: 1,581
	30 – 34: 2,133	30 – 34: 1,706	30 – 34: 2,266	30 – 34: 1,824	30 – 34: 2,295	30 – 34: 1,841
	35 – 39: 2,374	35 – 39: 2,122	35 – 39: 1,941	35 – 39: 1,615	35 – 39: 2,403	35 – 39: 2,006
	40 – 44: 2,390	40 – 44: 2,181	40 – 44: 1,557	40 – 44: 1,446	40 – 44: 2,299	40 – 44: 2,032
	45 – 49: 2,842	45 – 49: 2,853	45 – 49: 1,743	45 – 49: 1,717	45 – 49: 3,141	45 – 49: 2,784
	50 – 54: 2,955	50 – 54: 2,705	50 – 54: 1,751	50 – 54: 1,591	50 – 54: 3,791	50 – 54: 3,372
	55 – 59: 2,640	55 – 59: 2,296	55 – 59: 1,633	55 – 59: 1,361	55 – 59: 3,909	55 – 59: 3,169
	60 – 64: 1,871	60 – 64: 1,716	60 – 64: 1,069	60 – 64: 1,001	60 – 64: 2,970	60 – 64: 2,486
	65 – 69: 1,029	65 – 69: 1,062	65 – 69: 749	65 – 69: 656	65 – 69: 2,027	65 – 69: 2,010
	70 – 74: 1,212	70 – 74: 1,281	70 – 74: 643	70 – 74: 586	70 – 74: 1,918	70 – 74: 1,923
	75 – 79: 1,346	75 – 79: 1,405	75 – 79: 599	75 – 79: 617	75 – 79: 2,782	75 – 79: 2,752
	80 – 84: 1,510	80 – 84: 913	80 – 84: 325	80 – 84: 270	80 – 84: 2,916	80 – 84: 2,087
	85+: 1,510	85+: 913	85+: 325	85+: 270	85+: 2,916	85+: 2,087

Underestimation (Multiplication Factor)	Female<br><br>0: 35<br><br>1 – 4: 35<br><br>5 – 9: 51<br><br>10 – 14: 51<br><br>15 – 19: 121<br><br>20 – 24: 121<br><br>25 – 29: 121<br><br>30 – 34: 121<br><br>35 – 39: 106<br><br>40 – 44: 106<br><br>45 – 49: 106<br><br>50 – 54: 106<br><br>55 – 59: 106<br><br>60 – 64: 57<br><br>65 – 69: 57<br><br>70 – 74: 57<br><br>75 – 79: 57<br><br>80 – 84: 57<br><br>85+: 57<br><br>Average MF: 80.4	Male<br><br>0: 35<br><br>1 – 4: 35<br><br>5 – 9: 51<br><br>10 – 14: 51<br><br>15 – 19: 121<br><br>20 – 24: 121<br><br>25 – 29: 121<br><br>30 – 34: 121<br><br>35 – 39: 106<br><br>40 – 44: 106<br><br>45 – 49: 106<br><br>50 – 54: 106<br><br>55 – 59: 106<br><br>60 – 64: 57<br><br>65 – 69: 57<br><br>70 – 74: 57<br><br>75 – 79: 57<br><br>80 – 84: 57<br><br>85+: 57<br><br>Average MF: 80.4	Female<br><br>0: 35<br><br>1 – 4: 35<br><br>5 – 9: 43<br><br>10 – 14: 43<br><br>15 – 19: 83<br><br>20 – 24: 83<br><br>25 – 29: 83<br><br>30 – 34: 83<br><br>35 – 39: 86<br><br>40 – 44: 86<br><br>45 – 49: 86<br><br>50 – 54: 86<br><br>55 – 59: 86<br><br>60 – 64: 56<br><br>65 – 69: 56<br><br>70 – 74: 56<br><br>75 – 79: 56<br><br>80 – 84: 56<br><br>85+: 56<br><br>Average MF: 60	Male<br><br>0: 35<br><br>1 – 4: 35<br><br>5 – 9: 43<br><br>10 – 14: 43<br><br>15 – 19: 83<br><br>20 – 24: 83<br><br>25 – 29: 83<br><br>30 – 34: 83<br><br>35 – 39: 86<br><br>40 – 44: 86<br><br>45 – 49: 86<br><br>50 – 54: 86<br><br>55 – 59: 86<br><br>60 – 64: 56<br><br>65 – 69: 56<br><br>70 – 74: 56<br><br>75 – 79: 56<br><br>80 – 84: 56<br><br>85+: 56<br><br>Average MF: 60	Female<br><br>0: 68<br><br>1 – 4: 68<br><br>5 – 9: 53<br><br>10 – 14: 53<br><br>15 – 19: 82<br><br>20 – 24: 82<br><br>25 – 29: 82<br><br>30 – 34: 82<br><br>35 – 39: 83<br><br>40 – 44: 83<br><br>45 – 49: 83<br><br>50 – 54: 83<br><br>55 – 59: 83<br><br>60 – 64: 31<br><br>65 – 69: 31<br><br>70 – 74: 31<br><br>75 – 79: 31<br><br>80 – 84: 31<br><br>85+: 31<br><br>Average MF: 61.6	Male<br><br>0: 68<br><br>1 – 4: 68<br><br>5 – 9: 53<br><br>10 – 14: 53<br><br>15 – 19: 82<br><br>20 – 24: 82<br><br>25 – 29: 82<br><br>30 – 34: 82<br><br>35 – 39: 83<br><br>40 – 44: 83<br><br>45 – 49: 83<br><br>50 – 54: 83<br><br>55 – 59: 83<br><br>60 – 64: 31<br><br>65 – 69: 31<br><br>70 – 74: 31<br><br>75 – 79: 31<br><br>80 – 84: 31<br><br>85+: 31<br><br>Average MF: 61.6

Age distribution Data	Female	Male	Female	Male	Female	Male
	0: 348.856	0: 367,563	0: 362,170	0: 367,563	0: 384,735	0: 403,564
	1 – 4: 1,349,615	1 – 4: 1,420,377	1 – 4: 1,395,083	1 – 4: 1,420,377	1 – 4: 1,443,123	1 – 4: 1,525,024
	5 – 9: 1,669,514	5 – 9: 1,791,964	5 – 9: 1,736,124	5 – 9: 1,791,964	5 – 9: 1,756,737	5 – 9: 1,857,190
	10 – 14: 1,805,090	10 – 14: 1,903,744	10 – 14: 1,793,698	10 – 14: 1,903,744	10 – 14: 1,784,673	10 – 14: 1,893,522
	15 – 19: 1,972,135	15 – 19: 2,094,653	15 – 19: 2,000,494	15 – 19: 2,094,653	15 – 19: 1,985,471	15 – 19: 2,187,398
	20 – 24: 2,228,424	20 – 24: 2,357,904	20 – 24: 2,189,310	20 – 24: 2,357,904	20 – 24: 2,178,101	20 – 24: 2,395,930
	25 – 29: 2,514,805	25 – 29: 2,652,021	25 – 29: 2,588,966	25 – 29: 2,652,021	25 – 29: 2,579,651	25 – 29: 2,787,105
	30 – 34: 2,498,026	30 – 34: 2,576,055	30 – 34: 2,520,791	30 – 34: 2,576,055	30 – 34: 2,544,895	30 – 34: 2,676,180
	35 – 39: 2,359,171	35 – 39: 2,399,445	35 – 39: 2,444,464	35 – 39: 2,399,445	35 – 39: 2,500,432	35 – 39: 2,557,606
	40 – 44: 2,573,135	40 – 44: 2,611,144	40 – 44: 2,471,280	40 – 44: 2,611,144	40 – 44: 2,393,629	40 – 44: 2,428,357
	45 – 49: 3,318,228	45 – 49: 3,404,196	45 – 49: 3,221,159	45 – 49: 3,404,196	45 – 49: 3,097,169	45 – 49: 3,162,743
	50 – 54: 3,401,848	50 – 54: 3,454,804	50 – 54: 3,443,938	50 – 54: 3,454,804	50 – 54: 3,458,055	50 – 54: 3,526,252
	55 – 59: 2,937,399	55 – 59: 2,915,733	55 – 59: 3,026,149	55 – 59: 2,915,733	55 – 59: 3,118,379	55 – 59: 3,104,747
	60 – 64: 2,650,848	60 – 64: 2,502,129	60 – 64: 2,672,798	60 – 64: 2,502,129	60 – 64: 2,707,823	60 – 64: 2,573,457
	65 – 69: 2,082,925	65 – 69: 1,925,969	65 – 69: 2,251,562	65 – 69: 1,925,969	65 – 69: 2,376,693	65 – 69: 2,186,608
	70 – 74: 2,366,354	70 – 74: 2,059,860	70 – 74: 2,121,822	70 – 74: 2,059,860	70 – 74: 1,951,223	70 – 74: 1,703,714
	75 – 79: 2,297,800	75 – 79: 1,811,505	75 – 79: 2,380,229	75 – 79: 1,811,505	75 – 79: 2,418,877	75 – 79: 1,930,680
	80 – 84: 1,441,588	80 – 84: 955,114	80 – 84: 1,504,935	80 – 84: 955,114	80 – 84: 1,595,430	80 – 84: 1,099,541
	85+: 1,516,319	85+: 631,277	85+: 1,536,589	85+: 631,277	85+: 1,549,439	85+: 697,500

Life Expectancy Data	Female	Male	Female	Male	Female	Male
	0: 83.18	0: 78.36	0: 83.18	0: 78.36	0: 83.18	0: 78.36
	1 – 4: 79.48	1 – 4: 74.68	1 – 4: 79.48	1 – 4: 74.68	1 – 4: 79.48	1 – 4: 74.68
	5 – 9: 74.51	5 – 9: 69.71	5 – 9: 74.51	5 – 9: 69.71	5 – 9: 74.51	5 – 9: 69.71
	10 – 14: 69.54	10 – 14: 64.74	10 – 14: 69.54	10 – 14: 64.74	10 – 14: 69.54	10 – 14: 64.74
	15 – 19: 64.58	15 – 19: 59.81	15 – 19: 64.58	15 – 19: 59.81	15 – 19: 64.58	15 – 19: 59.81
	20 – 24: 59.64	20 – 24: 54.93	20 – 24: 59.64	20 – 24: 54.93	20 – 24: 59.64	20 – 24: 54.93
	25 – 29: 54.70	25 – 29: 50.06	25 – 29: 54.70	25 – 29: 50.06	25 – 29: 54.70	25 – 29: 50.06
	30 – 34: 49.78	30 – 34: 45.21	30 – 34: 49.78	30 – 34: 45.21	30 – 34: 49.78	30 – 34: 45.21
	35 – 39: 44.89	35 – 39: 40.41	35 – 39: 44.89	35 – 39: 40.41	35 – 39: 44.89	35 – 39: 40.41
	40 – 44: 40.05	40 – 44: 35.66	40 – 44: 40.05	40 – 44: 35.66	40 – 44: 40.05	40 – 44: 35.66
	45 – 49: 35.29	45 – 49: 31.03	45 – 49: 35.29	45 – 49: 31.03	45 – 49: 35.29	45 – 49: 31.03
	50 – 54: 30.65	50 – 54: 26.59	50 – 54: 30.65	50 – 54: 26.59	50 – 54: 30.65	50 – 54: 26.59
	55 – 59: 26.16	55 – 59: 22.42	55 – 59: 26.16	55 – 59: 22.42	55 – 59: 26.16	55 – 59: 22.42
	60 – 64: 21.85	60 – 64: 18.54	60 – 64: 21.85	60 – 64: 18.54	60 – 64: 21.85	60 – 64: 18.54
	65 – 69: 17.72	65 – 69: 14.94	65 – 69: 17.72	65 – 69: 14.94	65 – 69: 17.72	65 – 69: 14.94
	70 – 74: 13.79	70 – 74: 11.58	70 – 74: 13.79	70 – 74: 11.58	70 – 74: 13.79	70 – 74: 11.58
	75 – 79: 10.10	75 – 79: 8.49	75 – 79: 10.10	75 – 79: 8.49	75 – 79: 10.10	75 – 79: 8.49
	80 – 84: 6.99	80 – 84: 5.91	80 – 84: 6.99	80 – 84: 5.91	80 – 84: 6.99	80 – 84: 5.91
	85+: 2.24	85+: 2.02	85+: 2.24	85+: 2.02	85+: 2.24	85+: 2.02

	2018		2019		2020	
Average number of cases per year	Female	Male	Female	Male	Female	Male
	0: 1,517	0: 1,851	0: 1,480	0: 1,788	0: 1,522	0: 1,881
	1 – 4: 10,911	1 – 4: 12,235	1 – 4: 11,002	1 – 4: 12,161	1 – 4: 11,781	1 – 4: 13,143
	5 – 9: 10,661	5 – 9: 11,795	5 – 9: 7,186	5 – 9: 8,078	5 – 9: 10,331	5 – 9: 11,640
	10 – 14: 6,533	10 – 14: 7,182	10 – 14: 3,335	10 – 14: 3,843	10 – 14: 6,015	10 – 14: 7,206
	15 – 19: 5,662	15 – 19: 6,071	15 – 19: 3,580	15 – 19: 3,712	15 – 19: 4,844	15 – 19: 5,662
	20 – 24: 3,930	20 – 24: 3,294	20 – 24: 3,718	20 – 24: 3,002	20 – 24: 4,199	20 – 24: 3,629
	25 – 29: 4,684	25 – 29: 3,567	25 – 29: 5,000	25 – 29: 3,810	25 – 29: 5,775	25 – 29: 4,399
	30 – 34: 6,842	30 – 34: 5,197	30 – 34: 6,339	30 – 34: 5,245	30 – 34: 7,335	30 – 34: 6,121
	35 – 39: 9,654	35 – 39: 7,636	35 – 39: 6,378	35 – 39: 5,597	35 – 39: 6,604	35 – 39: 5,677
	40 – 44: 9,289	40 – 44: 7,932	40 – 44: 5,478	40 – 44: 5,112	40 – 44: 5,329	40 – 44: 4,889
	45 – 49: 10,831	45 – 49: 9,674	45 – 49: 5,829	45 – 49: 5,554	45 – 49: 5,093	45 – 49: 4,826
	50 – 54: 12,763	50 – 54: 11.,246	50 – 54: 7,325	50 – 54: 6,640	50 – 54: 5,715	50 – 54: 5,645
	55 – 59: 12,649	55 – 59: 10,530	55 – 59: 7,444	55 – 59: 6,378	55 – 59: 5,680	55 – 59: 4,974
	60 – 64: 9,451	60 – 64: 7,984	60 – 64: 5,590	60 – 64: 4,998	60 – 64: 4,217	60 – 64: 3,725
	65 – 69: 5,925	65 – 69: 5,478	65 – 69: 3,793	65 – 69: 3,677	65 – 69: 2,633	65 – 69: 2,561
	70 – 74: 4,764	70 – 74: 4,572	70 – 74: 3,121	70 – 74: 3,304	70 – 74: 2,069	70 – 74: 2,248
	75 – 79: 6,399	75 – 79: 5,787	75 – 79: 3,951	75 – 79: 3,929	75 – 79: 2,306	75 – 79: 2,309
	80 – 84: 5,543	80 – 84: 3,675	80 – 84: 4,174	80 – 84: 3,072	80 – 84: 2,649	80 – 84: 1,927
	85+: 5,543	85+: 3,675	85+: 4,174	85+: 3,072	85+: 2,649	85+: 1,927

Underestimation (Multiplication Factor)	Female<br><br>0: 30<br><br>1 – 4: 30<br><br>5 – 9: 30<br><br>10 – 14: 30<br><br>15 – 19: 50<br><br>20 – 24: 50<br><br>25 – 29: 50<br><br>30 – 34: 50<br><br>35 – 39: 40<br><br>40 – 44: 40<br><br>45 – 49: 40<br><br>50 – 54: 40<br><br>55 – 59: 40<br><br>60 – 64: 19<br><br>65 – 69: 19<br><br>70 – 74: 19<br><br>75 – 79: 19<br><br>80 – 84: 19<br><br>85+: 19<br><br>Average MF: 33.3	Male<br><br>0: 30<br><br>1 – 4: 30<br><br>5 – 9: 30<br><br>10 – 14: 30<br><br>15 – 19: 50<br><br>20 – 24: 50<br><br>25 – 29: 50<br><br>30 – 34: 50<br><br>35 – 39: 40<br><br>40 – 44: 40<br><br>45 – 49: 40<br><br>50 – 54: 40<br><br>55 – 59: 40<br><br>60 – 64: 19<br><br>65 – 69: 19<br><br>70 – 74: 19<br><br>75 – 79: 19<br><br>80 – 84: 19<br><br>85+: 19<br><br>Average MF: 33.3	Female<br><br>0: 19<br><br>1 – 4: 19<br><br>5 – 9: 17<br><br>10 – 14: 17<br><br>15 – 19: 22<br><br>20 – 24: 22<br><br>25 – 29: 22<br><br>30 – 34: 22<br><br>35 – 39: 26<br><br>40 – 44: 26<br><br>45 – 49: 26<br><br>50 – 54: 26<br><br>55 – 59: 26<br><br>60 – 64: 7<br><br>65 – 69: 7<br><br>70 – 74: 7<br><br>75 – 79: 7<br><br>80 – 84: 7<br><br>85+: 7<br><br>Average MF: 17.4	Male<br><br>0: 19<br><br>1 – 4: 19<br><br>5 – 9: 17<br><br>10 – 14: 17<br><br>15 – 19: 22<br><br>20 – 24: 22<br><br>25 – 29: 22<br><br>30 – 34: 22<br><br>35 – 39: 26<br><br>40 – 44: 26<br><br>45 – 49: 26<br><br>50 – 54: 26<br><br>55 – 59: 26<br><br>60 – 64: 7<br><br>65 – 69: 7<br><br>70 – 74: 7<br><br>75 – 79: 7<br><br>80 – 84: 7<br><br>85+: 7<br><br>Average MF: 17.4	Female<br><br>0: 19<br><br>1 – 4: 19<br><br>5 – 9: 17<br><br>10 – 14: 17<br><br>15 – 19: 22<br><br>20 – 24: 22<br><br>25 – 29: 22<br><br>30 – 34: 22<br><br>35 – 39: 26<br><br>40 – 44: 26<br><br>45 – 49: 26<br><br>50 – 54: 26<br><br>55 – 59: 26<br><br>60 – 64: 7<br><br>65 – 69: 7<br><br>70 – 74: 7<br><br>75 – 79: 7<br><br>80 – 84: 7<br><br>85+: 7<br><br>Average MF: 17.4	Male<br><br>0: 19<br><br>1 – 4: 19<br><br>5 – 9: 17<br><br>10 – 14: 17<br><br>15 – 19: 22<br><br>20 – 24: 22<br><br>25 – 29: 22<br><br>30 – 34: 22<br><br>35 – 39: 26<br><br>40 – 44: 26<br><br>45 – 49: 26<br><br>50 – 54: 26<br><br>55 – 59: 26<br><br>60 – 64: 7<br><br>65 – 69: 7<br><br>70 – 74: 7<br><br>75 – 79: 7<br><br>80 – 84: 7<br><br>85+: 7<br><br>Average MF: 17.4

Age distribution Data	Female	Male	Female	Male	Female	Male
	0: 382,505	0: 402,569	0: 381,896	0: 402,082	0: 377,310	0: 397,560
	1 – 4: 1,490,055	1 – 4: 1,571,649	1 – 4: 1,530,404	1 – 4: 1,612,015	1 – 4: 1,552,357	1 – 4: 1,634,149
	5 – 9: 1,770,798	5 – 9: 1,871,418	5 – 9: 1,780,063	5 – 9: 1,882,175	5 – 9: 1,812,127	5 – 9: 1,914,843
	10 – 14: 1,787,506	10 – 14: 1,895,259	10 – 14: 1,797,750	10 – 14: 1,904,430	10 – 14: 1,797,762	10 – 14: 1,905,151
	15 – 19: 1,949,255	15 – 19: 2,131,312	15 – 19: 1,920,778	15 – 19: 2,082,699	15 – 19: 1,900,397	15 – 19: 2,038,846
	20 – 24: 2,189,438	20 – 24: 2,413,076	20 – 24: 2,192,260	20 – 24: 2,415,012	20 – 24: 2,191,934	20 – 24: 2,416,497
	25 – 29: 2,548,258	25 – 29: 2,749,822	25 – 29: 2,497,399	25 – 29: 2,695,936	25 – 29: 2,438,489	25 – 29: 2,635,982
	30 – 34: 2,573,555	30 – 34: 2,716,697	30 – 34: 2,624,948	30 – 34: 2,784,081	30 – 34: 2,665,455	30 – 34: 2,830,265
	35 – 39: 2,550,417	35 – 39: 2,612,793	35 – 39: 2,584,099	35 – 39: 2,653,317	35 – 39: 2,606,089	35 – 39: 2,683,121
	40 – 44: 2,377,131	40 – 44: 2,411,226	40 – 44: 2,402,780	40 – 44: 2,438,958	40 – 44: 2,438,092	40 – 44: 2,470,934
	45 – 49: 2,944,060	45 – 49: 2,999,227	45 – 49: 2,772,302	45 – 49: 2,812,217	45 – 49: 2,624,418	45 – 49: 2,648,940
	50 – 54: 3,450,056	50 – 54: 3,517,989	50 – 54: 3,405,726	50 – 54: 3,470,222	50 – 54: 3,326,464	50 – 54: 3,383,771
	55 – 59: 3,205,222	55 – 59: 3,196,294	55 – 59: 3,300,544	55 – 59: 3,297,674	55 – 59: 3,366,915	55 – 59: 3,370,390
	60 – 64: 2,747,063	60 – 64: 2,627,990	60 – 64: 2,798,189	60 – 64: 2,695,725	60 – 64: 2,868,318	60 – 64: 2,779,453
	65 – 69: 2,459,543	65 – 69: 2,252,151	65 – 69: 2,516,863	65 – 69: 2,291,634	65 – 69: 2,546,505	65 – 69: 2,312,527
	70 – 74: 1,927,417	70 – 74: 1,684,492	70 – 74: 1,916,659	70 – 74: 1,679,886	70 – 74: 1,955,815	70 – 74: 1,718,280
	75 – 79: 2,350,988	75 – 79: 1,884,435	75 – 79: 2,265,773	75 – 79: 1,823,611	75 – 79: 2,143,390	75 – 79: 1,733,030
	80 – 84: 1,698,543	80 – 84: 1,186,669	80 – 84: 1,824,473	80 – 84: 1,287,124	80 – 84: 1,923,991	80 – 84: 1,370,290
	85+: 1,546,976	85+: 718,497	85+: 1,539,616	85+: 737,893	85+: 1,923,991	85+: 793,584

Life Expectancy Data	Female	Male	Female	Male	Female	Male
	0: 83.40	0: 83.40	0: 83.40	0: 83.40	0: 83.40	0: 83.40
	1 – 4: 79.68	1 – 4: 79.68	1 – 4: 79.68	1 – 4: 79.68	1 – 4: 79.68	1 – 4: 79.68
	5 – 9: 74.71	5 – 9: 74.71	5 – 9: 74.71	5 – 9: 74.71	5 – 9: 74.71	5 – 9: 74.71
	10 – 14: 69.73	10 – 14: 69.73	10 – 14: 69.73	10 – 14: 69.73	10 – 14: 69.73	10 – 14: 69.73
	15 – 19: 64.77	15 – 19: 64.77	15 – 19: 64.77	15 – 19: 64.77	15 – 19: 64.77	15 – 19: 64.77
	20 – 24: 59.82	20 – 24: 59.82	20 – 24: 59.82	20 – 24: 59.82	20 – 24: 59.82	20 – 24: 59.82
	25 – 29: 54.88	25 – 29: 54.88	25 – 29: 54.88	25 – 29: 54.88	25 – 29: 54.88	25 – 29: 54.88
	30 – 34: 49.96	30 – 34: 49.96	30 – 34: 49.96	30 – 34: 49.96	30 – 34: 49.96	30 – 34: 49.96
	35 – 39: 45.07	35 – 39: 45.07	35 – 39: 45.07	35 – 39: 45.07	35 – 39: 45.07	35 – 39: 45.07
	40 – 44: 40.23	40 – 44: 40.23	40 – 44: 40.23	40 – 44: 40.23	40 – 44: 40.23	40 – 44: 40.23
	45 – 49: 35.46	45 – 49: 35.46	45 – 49: 35.46	45 – 49: 35.46	45 – 49: 35.46	45 – 49: 35.46
	50 – 54: 30.08	50 – 54: 30.08	50 – 54: 30.08	50 – 54: 30.08	50 – 54: 30.08	50 – 54: 30.08
	55 – 59: 26.29	55 – 59: 26.29	55 – 59: 26.29	55 – 59: 26.29	55 – 59: 26.29	55 – 59: 26.29
	60 – 64: 21.96	60 – 64: 21.96	60 – 64: 21.96	60 – 64: 21.96	60 – 64: 21.96	60 – 64: 21.96
	65 – 69: 17.83	65 – 69: 17.83	65 – 69: 17.83	65 – 69: 17.83	65 – 69: 17.83	65 – 69: 17.83
	70 – 74: 13.93	70 – 74: 13.93	70 – 74: 13.93	70 – 74: 13.93	70 – 74: 13.93	70 – 74: 13.93
	75 – 79: 10.28	75 – 79: 10.28	75 – 79: 10.28	75 – 79: 10.28	75 – 79: 10.28	75 – 79: 10.28
	80 – 84: 7.08	80 – 84: 7.08	80 – 84: 7.08	80 – 84: 7.08	80 – 84: 7.08	80 – 84: 7.08
	85+: 2.15	85+: 2.15	85+: 2.15	85+: 2.15	85+: 2.15	85+: 2.15

Supplementary Table 4: Scenario Input Parameters

Sequelae	Probability	Disability weight	Duration (in years)	Source
Pneumonia	Lower bound: 0.016<br>Baseline: 0.016<br>Upper bound: 0.036	RandPert(0.051, 0.1, 0.133)	0.04	IHME 2019<br>Rapid Review
Pneumonia death	Lower bound: 0.16<br>Baseline: 0.16<br>Upper bound: 0.16	-	-	Arias-Fernandez et al., 2021<br>Boyd et al., 2006<br>Theilacker et al., 2021<br>Topoulus et al., 2019
Bronchitis	Lower bound: 0.0213<br>Baseline: 0.0213<br>Upper bound: 0.0213	RandUniform(0.051, 0.133)	0.04	IHME 2019<br>Rapid Review
Chronic Bronchitis	Lower bound: 0.028<br>Baseline: 0.028<br>Upper bound: 0.028	RandPert(0.019, 0.225, 0.408)	Rem. Life exp.	IHME 2019<br>Edmond et al., 2012
Otitis Media	Lower bound: 0.0065<br>Baseline: 0.0151<br>Upper bound: 0.0151	RandPert(0.007, 0.013, 0.024)	RandUniform(0.038, 0.057)	IHME 2019<br>Wielders et al., 2010<br>Monasta et al., 2012
Deafness	Lower bound: 0.00006<br>Baseline: 0.00006<br>Upper bound: 0.00006	0.23	Rem. Life exp.	Meier et al., 2000<br>Wielders et al., 2010<br>Plass et al., 2014
Encephalitis	Lower bound: 0.002<br>Baseline: 0.002<br>Upper bound: 0.002	RandPert(0.088, 0.133, 0.19)	0.06	IHME 2019<br>Rapid Review
Encephalitis death	Lower bound: RandUniform(0.041, 0.056)<br>Baseline: 0.16<br>Upper bound: 0.16	-	-	George et al., 2014<br>Hjalmarsson et al., 2009
Myocarditis	Lower bound: 0.001		0.04	IHME 2019

Sequelae	Probability	Disability weight	Duration (in years)	Source
	Baseline: 0.001<br>Upper bound: 0.001	RandPert(0.032, 0.051, 0.074)		Rapid Review
Myocarditis death	Lower bound: 0.081<br>Baseline: 0.2<br>Upper bound: 0.2	-	-	Kang & An, 2021<br>Kuehl & Schultheiss, 2012
ARDS	Lower bound: 0.00023<br>Baseline: 0.00023<br>Upper bound:<br>0 – 4: 0.0019<br>5 – 34: 0.0078<br>35 – 59: 0.003<br>60 – 79: 0.01<br>80 – 85+: 0.02	RandPert(0.019, 0.225, 0.408)	0.09	IHME 2019<br>Wielders et al., 2010
ARDS death	Lower bound: 0.35<br>Baseline: 0.4<br>Upper bound: 0.4	-	-	Lewis et al., 2019
Permanent disability due to ARDS	Lower bound: 0.56<br>Baseline: 0.56<br>Upper bound: 0.56	0.18	Rem. Life exp.	Stouthard et al., 1997
Sepsis	Lower bound: 0.000097<br>Baseline: 0.000097<br>Upper bound: 0.000097	RandPert(0.579, 0.655, 0.727)	RandUniform(0.027, 0.038)	IHME 2019<br>Cassini et al., 2018
Sepsis death	Lower bound: RandPert(0.198, 0.22, 0.243)<br>Baseline: RandPert(0.198, 0.22, 0.243)<br>Upper bound: RandPert(0.198, 0.22, 0.243)	-	-	
	Lower bound: 0.17<br>Baseline: 0.17	0.28	Rem. Life exp.	Plass et al., 2014

Sequelae	Probability	Disability weight	Duration (in years)	Source
Permanent disability due to sepsis	Upper bound: 0.17			
Sinusitis	Lower bound: 0.0024	RandUniform(0.051, 0.133)	0.04	IHME 2019
	Baseline: 0.0024			
	Upper bound: 0.0024			
Death (for previous modeling approach only, see Supplement 6)	Lower bound: 0.001	-	-	Cassini et al., 2018<br>Rapid Review
	Baseline: 0.001			
	Upper bound: 0.0156			
Symptomatic infection	-	RandPert(0.007, 0.051, 0.125)	0.01	IHME 2019
